# Subgingival microbiome of experimental gingivitis: shifts associated with the use of chlorhexidine and *N*-acetyl cysteine mouthwashes

**DOI:** 10.1080/20002297.2019.1608141

**Published:** 2019-06-24

**Authors:** Ahlam Al-Kamel, Divyashri Baraniya, Wadhah Abdulnaser Al-Hajj, Esam Halboub, Saleem Abdulrab, Tsute Chen, Nezar Noor Al-Hebshi

**Affiliations:** a Department of Preventive and Biomedical Science, Faculty of Dentistry, University of Science and Technology, Sanaa, Yemen; b Oral Microbiome Research Laboratory, Maurice H. Kornberg School of Dentistry, Temple University, Philadelphia, PA, USA; c Department of Periodontology, Faculty of Dentistry, Thamar University, Thamar, Yemen; d Department of Maxillofacial Surgery and Diagnostic Sciences, College of Dentistry, Jazan University, Jazan, Saudi Arabia; e Madinat Khalifa Health Center, Primary Health Care Corporation, Doha, Qatar; f Department of Microbiology, Forsyth Institute, Cambridge, MA, USA

**Keywords:** 16S rRNA, bacteria, gingivitis, high-throughput nucleotide sequencing, microbiota, mouthwashes

## Abstract

This study aimed to demonstrate subgingival microbial changes associated with development, prevention, and treatment of experimental gingivitis using chlorhexidine (CHX) and *N*-acetylcysteine (NAC) mouthwashes. This randomized clinical trial comprised two parts: a 3-week prevention sub-study in which 30 study subjects were equally assigned to either mouthwash or placebo while developing experimental gingivitis; followed by a 2-week treatment sub-study in which 20 subjects with experimental gingivitis were assigned to either mouthwash. Subgingival samples were collected at the beginning and end of each sub-study for microbial profiling with *16S rRNA* gene sequencing. As expected, CHX was effective in both preventing and reversing experimental gingivitis; NAC had a modest effect. Gingivitis was associated with enrichment of TM7 HOT-346/349, *Tannerella* HOT-286, *Cardiobacterium valvarum, Campylobacter gracilis, Porphyromonas catoniae, Leptotrichia* HOT-219, and *Selenomonas* spp. At the phylum/genus level, TM7 showed the strongest association. Gingival health was associated with increased abundance of *Haemophilus parainfluenzae, Lautropia mirabilis, Rothia* spp., *Streptococcus* spp., and *Kingella oralis*. CHX demonstrated largely indiscriminate antimicrobial action, resulting in significant drop in biomass and diversity. Our results substantiate the role of specific oral bacterial species in the development of gingivitis. They also indicate that NAC is not a promising mouthwash at the concentration tested.

Gingivitis is a reversible inflammation of the gingiva; the most common form of it is ‘plaque-induced gingivitis’ which is caused by dental biofilm accumulation below and around the gingival margin [1]. Typically, plaque-induced gingivitis is painless and is associated with subtle clinical changes, such as gingival redness and oedema, that are frequently unnoticed by the patients making them unaware of the disease [2]. In more severe forms of the disease, the patient may notice bleeding on brushing, gingival swelling, and even halitosis. Although usually not associated with bone loss, plaque-induced gingivitis is a prerequisite for progression to periodontitis, and thus, its management is a cornerstone in the prevention of periodontitis [1].

In the mid-1960s, Loe and co-workers were the first to experimentally establish the role of quantity (biomass) and quality (microbial composition) of dental plaque in the aetiology gingivitis [3,4]. Over the following three decades, the microbiology of naturally occurring as well as experimental gingivitis was extensively studied in both children and adults, primarily using culture techniques [5–11]. Morphologically, transition to gingivitis was found to be associated with a decrease in Gram-positive cocci and rods, and an increase in Gram-negative cocci and rods, filamentous bacteria, and spirochetes [4]. Despite methodological variations, a number of species were quite consistently found to be associated with gingivitis in those studies, including *Actinomyces viscosus, Actinomyces israelii, Fusobacterium nucleatum, Selenomonas* spp., *Eubacterium* spp., *Campylobacter* spp., *Prevotella* spp., and *Treponema* spp. [5–11]. It was also observed that bacterial diversity increased with development and progression of gingivitis [8]. Interest in the microbiology of gingivitis declined for over a decade after that but has re-emerged recently with the introduction of the concept of microbiome and the advent of molecular technologies such as next generation technologies (NGS) – developments that revolutionized the study of microbial communities.

Unlike the case with periodontitis, using NGS analysis of the *16S rRNA* gene to study the microbiome associated with naturally occurring and experimental gingivitis is limited to a few studies by Kistler et al. and Huang et al. [12–14]. In addition to substantiating the potential role of some taxa identified in early studies, results from these studies provide the evidence for involvement of a more complex microbial consortium including species of the genera *Tannerella, Porphyromonas, Lachnospiraceae*, and those belonging to the new phyla *Saccharibacteria* (TM7) and SR-1. In fact, Huang et al. [12] developed a microbial index based on abundance of 27 genera (MiG27) that could differentiate between healthy and gingivitis state in a validation cohort with 95% accuracy. In a later study, Huang et al. [15] used the same approach to characterize the microbial shifts associated with anti-gingivitis treatments, including a comparison between tooth brushing alone and combining it with a cetylpyridinium chloride-based mouthwash. With the exception of this study, our understanding of the impact of different anti-gingivitis treatments on the oral microbiome remains limited; this applies even to chlorhexidine (CHX), the most commonly used mouthwash for plaque control. Also, the microbiome changes associated with gingivitis reported by Huang et al. need to be replicated by independent research groups.

*N*-acetyl cysteine (NAC) is a compound with antibacterial and antioxidant properties that has received considerable attention recently [16]. *In vitro*, NAC has been found to inhibit biofilm formation by medically important bacteria as well as oral pathogens [17–21]; it has also been shown to reduce biomass and viability of a multispecies, plaque-derived biofilm [22]. In a very recent clinical trial [23], we have compared the clinical efficacy of a NAC-based mouthwash in the prevention and treatment of experimental gingivitis as compared to CHX. This study is a sub-study of that clinical trial, in which we aimed to (1) demonstrate subgingival microbiome changes associated with the development of experimental gingivitis and (2) characterize the microbiome shifts induced by the use of 1.25% NAC mouthwash for prevention and treatment of gingivitis as compared to 0.2% CHX.

## Materials and methods

### Study design and population

**Figure 1.**
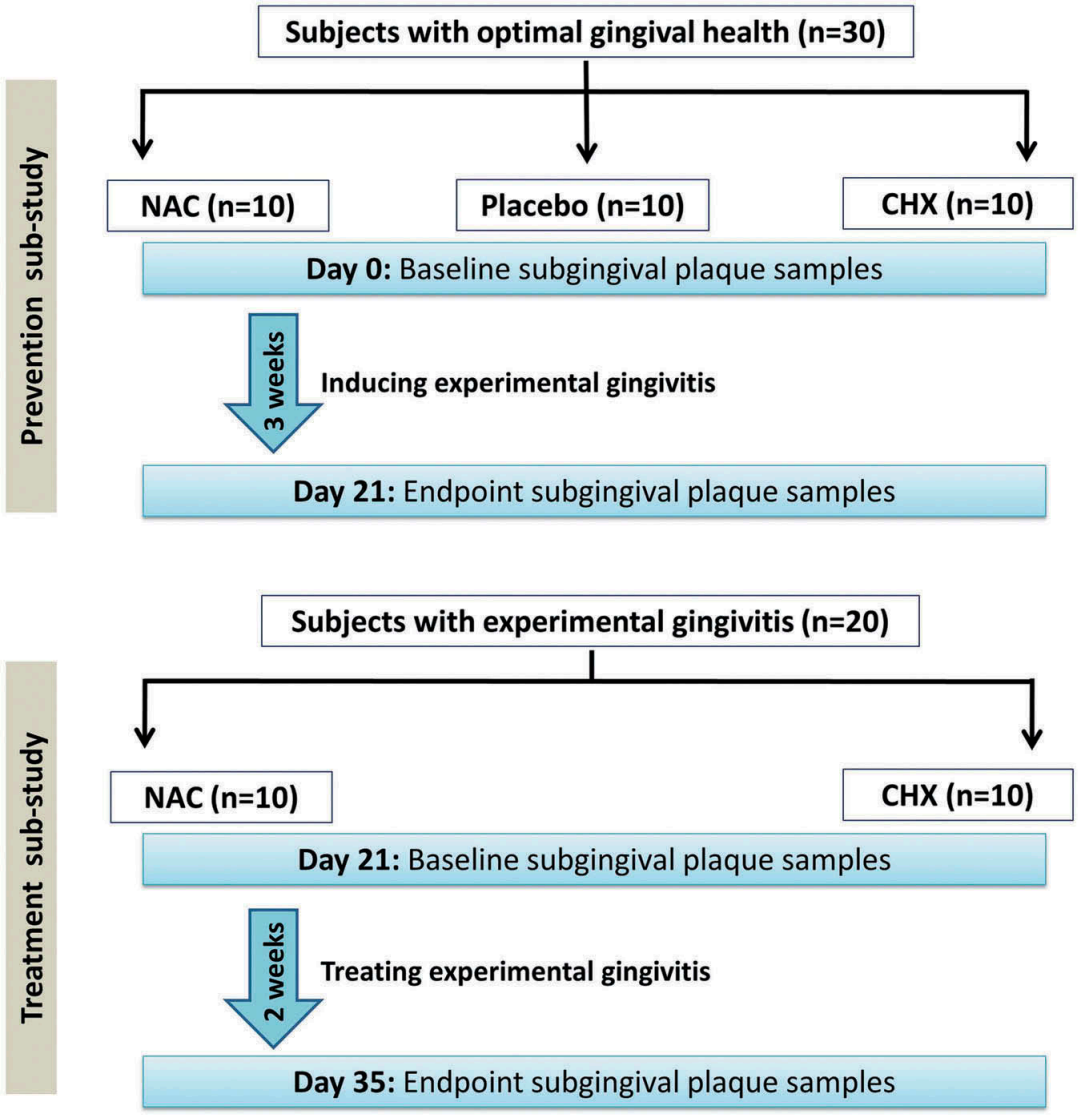
A flowchart diagram showing the study design. A detailed CONSORT flowchart of the parent clinical trial can be found in the original publication [23].

This was a sub-study of a larger randomized, triple-blinded, placebo-controlled, parallel-arm clinical trial, in which 1.25% NAC and 0.2% CHX were compared for effectiveness in prevention and treatment of full-mouth experimental gingivitis. Details about the study design, sampling, inclusion and exclusion criteria, randomization, preparation and administration of the mouthwashes, clinical measurements, and statistical analyses can be found in the original publication [23]. A summary of the design of the current microbiome study is provided in Figure 1, and the characteristics of the study subjects included are presented in Supplementary Tables 1 and 2. In brief, this study was conducted in two parts: prevention and treatment sub-studies. In the former, 30 subjects brought to optimal gingival health were randomly and equally allocated into either NAC, CHX, or placebo (base formula with no active ingredient) for 3 weeks, during which they were instructed to refrain from other oral hygiene measures to induce experimental gingivitis. In the treatment sub-study, 20 subjects who developed experimental gingivitis at the end of the 3 weeks (the 10 subjects in the placebo group above plus another 10 from the larger clinical trial) were assigned equally to either mouthwash and followed for additional 2 weeks. Collection of subgingival plaque samples in addition to measuring plaque index, gingival index, and papillary bleeding index was performed at the beginning and end of each sub-study.

### Sampling of subgingival plaque and DNA extraction

For each subject, subgingival plaque was sampled from the buccal sulcus of the first molar in each quadrant. The sites were isolated with sterile cotton rolls and dried gently with air spray in order to avoid contamination with saliva. Supragingival plaque and debris were removed before sampling. For each site, a sterile, size 30 paper point was inserted as deep as possible into the gingival sulcus mesiobuccally and passed along the sulcus to the most distobuccal point; the paper points from the four sites were then pooled into a tube containing 750 μl Tris-EDTA buffer (pH 8) and stored at −20°C. One tube with buffer and sterile paper points was used as a negative control throughout downstream analysis.

Prior to DNA extraction, the samples were thawed, vortexed vigorously to detach dental plaque from the paper points into the TE buffer, and centrifuged at 16,000 *g* for 5 min to pellet the cells. The pellets were each washed twice in 1 ml phosphate buffer saline (pH 7.5), suspended in 180 μl of the digestion buffer (25 mM Tris-HCl, pH 8.0, 2.5 mM EDTA, 1% Triton X-100) containing 20 mg/ml lysozyme, and incubated at 37°C overnight. DNA was then extracted using the QIAamp genomic DNA kit (Qiagen, Germany) according to the manufacturer’s instructions; 200 μl of the AE buffer provided in the kit was used for elution. The quantity of DNA was assessed by Qubit® 2.0 Fluorometer (Life Technologies, USA). The extracts were stored at −20°C for subsequent analysis.

### Amplicon library preparation and sequencing

This was performed as described by Illumina (manual 15044223 Rev. B) and according to the details provided elsewhere [24]. Briefly, the degenerate primers 27FYM [25] and 519R [26], linked to Illumina’s specific adapter sequences, were used to amplify the V1-3 region of the 16S rRNA gene, in standard Polymerase Chain Reaction (PCR) conditions. The amplicons resulting from this step (~520 bp) were purified using Agencourt AMPure XP beads (Beckman Coulter, USA) and then indexed in a second PCR using the Nextera XT v2 Index Kit (Illumina, USA). Indexed amplicons were combined together in equimolar concentrations and sequenced on the MiSeq Sequencing System (Illumina, USA) using v3 2 × 300 bp, paired-end sequencing chemistry at the CHOP Microbiome (University of Pennsylvania, USA).

### Preprocessing of sequencing data

Forward and reverse paired-end reads were merged with PEAR [27] using the following parameters: minimum length = 460 bp, maximum length = 570 bp, minimum overlap = 30 bp, and *p*-value = 0.001. Merged reads were quality-filtered using MOTHUR [28] as follows: reads with primer mismatches, homopolymers longer than eight bases or ambiguous bases were excluded; the remaining reads were then screened using a sliding, 50-bp window with average *Q* score of 35, discarding reads shorter than 420 bp. Sequences were aligned against the Silva ribosomal database for bacteria [29], and those with poor alignment were removed. Chimeric sequences were detected and filtered out applying the UCHIME algorithm [30] in MOTHUR, using self-reference [31]. Preliminary taxonomy assignment was preformed using the Wang’s Bayesian classifier and Green genes bacterial database [32] as a reference to identify and hence exclude sequences from rare phyla not typically found in the oral cavity, as well as contaminants found in the negative control.

### Compositional data analysis

The high-quality, non-chimeric sequences were classified to the species level using the prioritized, BLASTN-based taxonomy assignment algorithm described by Al-hebshi et al. [33] and as implemented in a later study [34]. In brief, reads were individually searched at an alignment coverage and % identity of ≥98% against four sets of *16S rRNA* reference sequences: The Human Oral Microbiome Database (HOMD) version 14.5; a chimera-free version of the Human Oral Microbiome extended database (trusted-HOMDext); a modified version of the Greengene Gold set (modified-GGG); and NCBI’s Microbial 16S set. Reads were each assigned species taxonomy of the top hit reference sequence: the sequence with the highest % identity and bit score belonging to the highest priority reference set. Reads with multiple hits were assigned multiple-species taxonomies while reads with no matches were *de novo* clustered into an operational taxonomy unit (OTU) calling at 98% identity cut-off using USEARCH [35]. OTUs with less than 100 sequences were excluded while the rest was assigned to the closest species from the four databases and considered potentially novel species. More details about the algorithm including taxonomy assignment and database prioritization can be found in the original publications [33,34].

Subsampling, generation of taxonomy plots/tables and rarefaction curves, and calculation of species richness, coverage, and alpha and beta diversity indices were carried out in QIIME [36]. The study subjects were clustered with principle component analysis (PCoA) based on abundance Jaccard distance metric. Linear discriminant analysis (LDA) effect size (LEfSe) [37] was performed to detect differentially abundant taxa between the groups.

## Results

### Sequencing and data preprocessing statistics

The raw sequence data reported in this paper have been deposited in the Genome Sequence Archive [38] of the BIG Data Center [39], Beijing Institute of Genomics, Chinese Academy of Sciences, under accession no. CRA001406 that is publicly accessible at http://bigd.big.ac.cn/gsa. A total of 11,998,378 raw reads were obtained from the 90 samples, ranging 66,689–240,851 reads per sample (Supplementary file 1). Around 98% of the reads were successfully stitched; however, only 39.31% of them were retained after quality filtration. Filtering out misaligned, chimeric, and contaminant sequences resulted in the removal of additional 18% of the reads, leaving behind a total of 2575,688 high-quality, non-chimeric merged reads (21.47% of the raw data) with an average of length of ~480 bp. Nearly 90% of these reads could be assigned a taxonomy: the number of classified reads per sample ranged from 8967 to 53,358 reads (average 25,281 ± 8917).

### Clinical findings

**Figure 2.**
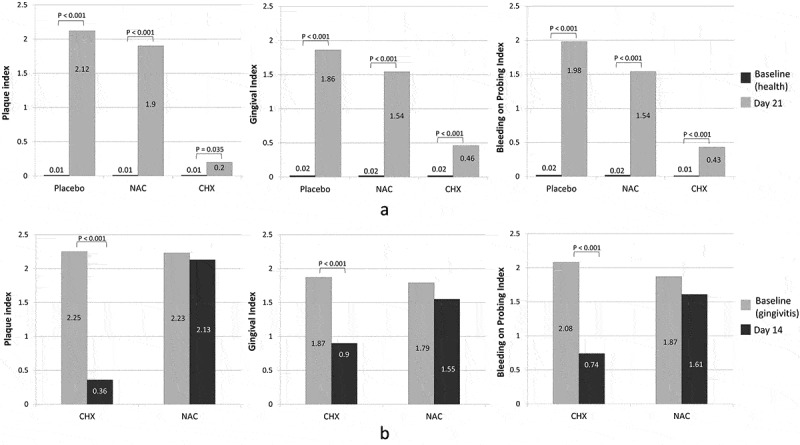
Within-group changes in gingival health status from baseline to endpoint in the (a) prevention sub-study and (b) treatment sub-study. NAC: *N*-acetyl cysteine; CHX: chlorhexidine. Significance of within-group differences were sought using Wilcoxon signed-rank test.

The changes from baseline to endpoint of gingival health status in each of the two sub-studies are shown in Figure 2. In the prevention sub-study, there was significant increase in all indices from Day 0 (health) to Day 21 (experimental gingivitis). However, the increases were considerably lower in the CHX group compared to the other groups, i.e. CHX was not able to completely prevent changes in clinical parameters. The differences in changes between the placebo and NAC groups were not statistically significant (Supplementary Table 3). In the treatment sub-study, CHX was associated with a remarkable reduction in plaque and gingivitis, although it did not restore clinical parameters to baseline, while NAC resulted in insignificant reductions; the differences in changes between the two groups were statistically significant (Supplementary Table 4).

### General microbiological findings

**Figure 3.**
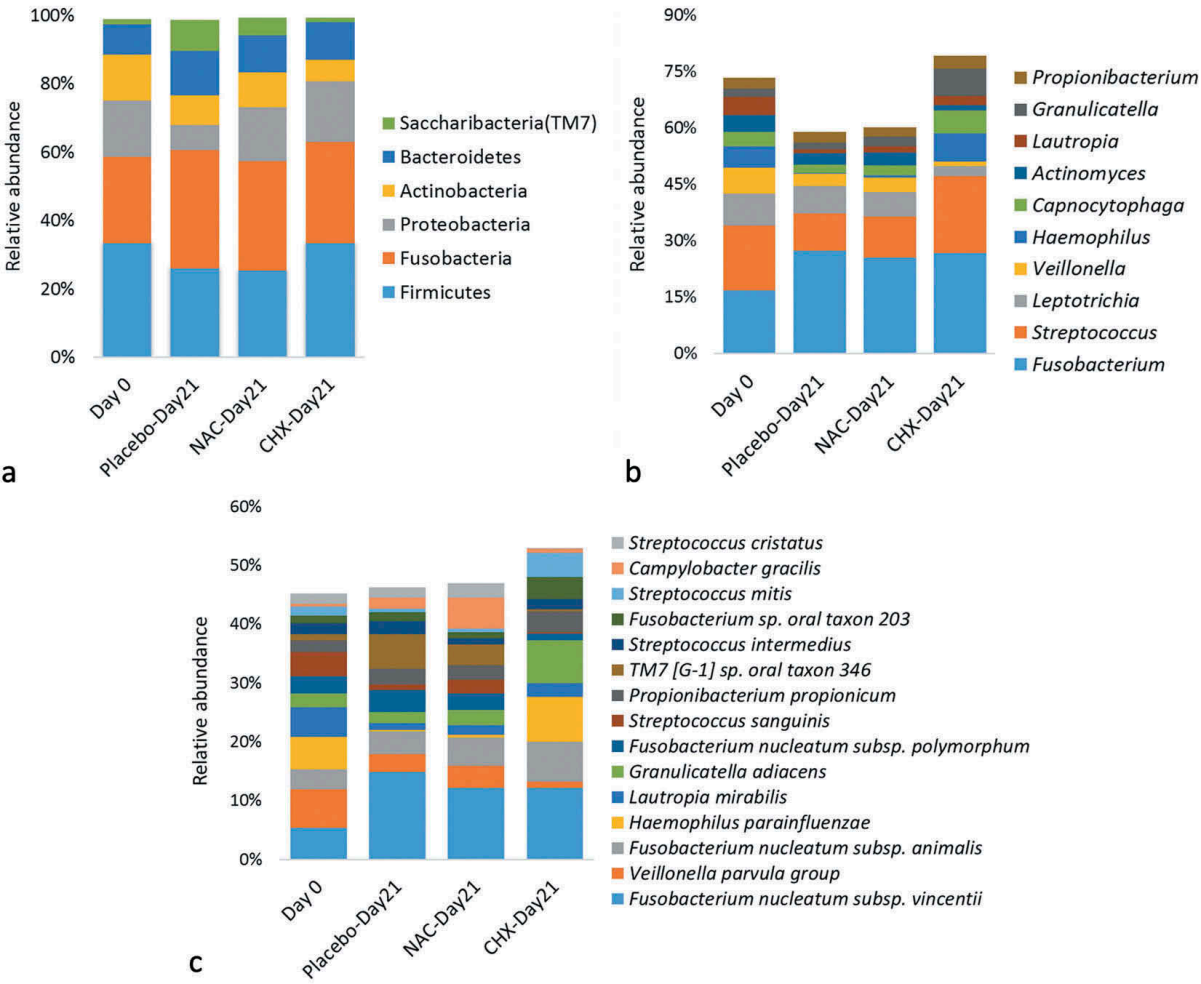
Summary taxonomy profiles in the prevention sub-study. The relative abundances of bacterial phyla with >1% abundance, 10 most abundant genera, and 15 most abundant species. For the purpose of this descriptive figure, baseline (heathy) samples from all three groups were pooled.

**Figure 4.**
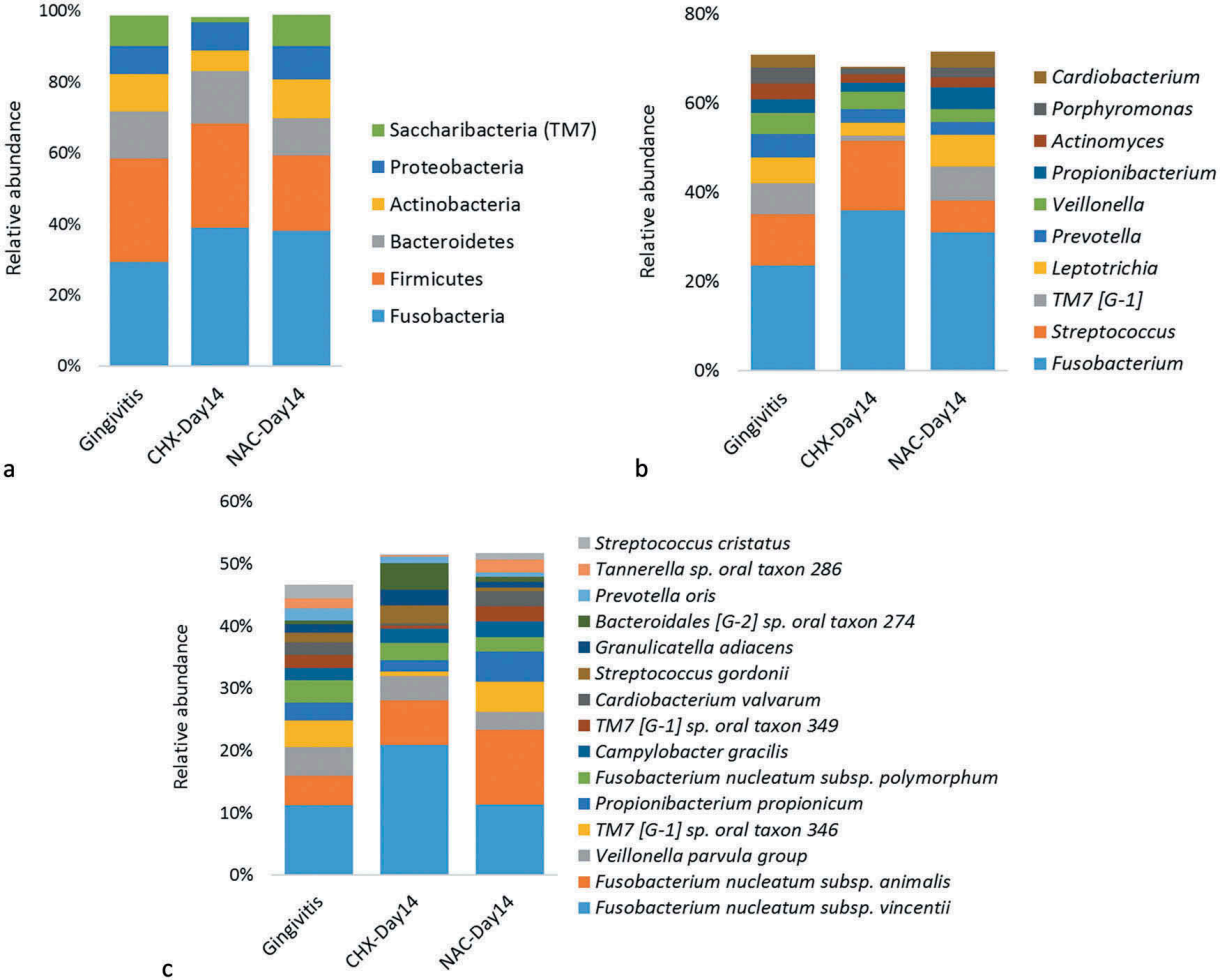
Summary taxonomy profiles in the treatment sub-study. The relative abundances of bacterial phyla with >1% abundance, 10 most abundant genera, and 15 most abundant species. For the purpose of this descriptive figure, baseline (gingivitis) samples from the two groups were pooled.

A total of 785 species – 34 of which potentially novel – belonging to 219 genera and 11 bacterial phyla were identified overall. The relative abundances and detection frequencies of taxa at the three taxonomic levels for each sample/group in the two sub-studies are provided in Supplementary files 2–7. The number of taxa per sample ranged from 39 to 84 genera and from 70 to 284 species. The relative abundances of bacterial phyla with >1% abundance (which together constituted more than 99% of all identified phyla), the 10 most abundant genera, and the 15 most abundant species are presented in Figures 3 and 4. Overall, *Fusobacterium, Streptococcus, Leptotrichia, Veillonella, Propionibacterium*, and *Actinomyces* were among the most abundant genera regardless of the time points or interventions; at the species levels were *F. nucleatum*, the *Veillonella parvula* group, *Propionibacterium propionicum*, and *Granulicatella adiacens*.

### Species richness, diversity, and coverage

**Figure 5.**
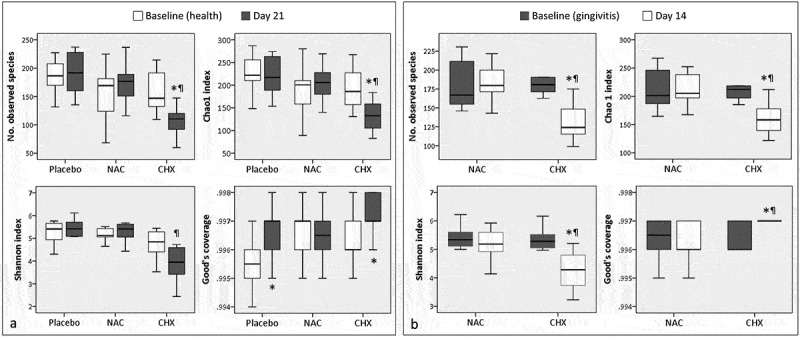
Box and whisker plots of observed and expected (Chao1) species richness, alpha diversity (Shannon index), and coverage (Good’s) at baseline and endpoint in each group. (a) Prevention sub-study; (b) Treatment sub-study. *Statistically significant difference compared to baseline (within-group); Wilcoxon signed-rank test. ^¶^Statistically significant difference compared to the corresponding time point in the other group(s); Mann–Whitney *U* test.

The changes in observed and expected species richness and alpha diversity (Shannon index) associated with development of experimental gingivitis and the use of the mouthwashes are illustrated in Figure 5. At Day 21 of the prevention sub-study, the placebo and NAC groups (i.e. groups that developed full-blown gingivitis) showed a slight, but not significant, increase in species richness and alpha diversity compared to Day 0 (healthy gingiva), while the use of CHX resulted in a significant drop in these parameters. In the treatment sub-study, CHX was also associated with a significant decrease in species richness and alpha diversity, while using NAC did not result in any significant changes. Adequate species coverage was obtained (≥99.4%) for all samples, but those in the CHX groups had significantly higher coverage.

**Figure 6.**
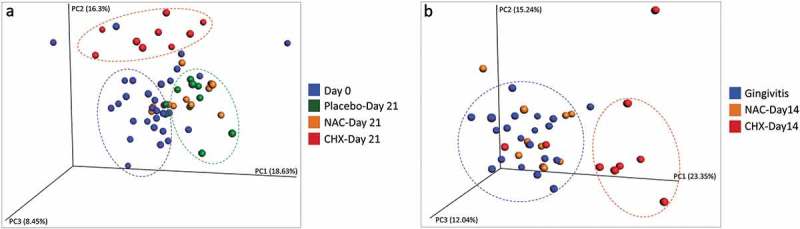
Principle component (PCoA) analysis plots for the (a) prevention sub-study and (b) treatment sub-study. Samples were clustered based on abundance-Jaccard distance matrix.

The results of PCoA for the prevention sub-study are presented in Figure 6(a). The plot shows the first three principle components to account for 43.38% of all variation in the microbiome among the samples. Three major clusters formed. One cluster comprised mainly samples associated with healthy gingiva (Day 0) from all three groups, while the second cluster included those from the CHX group on Day 21. Samples from the placebo and NAC groups on Day 21 (i.e. those with severe gingivitis) are clustered together. Figure 6(b) shows the PCoA plot for the treatment sub-study in which more than 50% of variation was explained by principle components 1, 2, and 3. The Day 14 samples from the NAC group co-clustered with the gingivitis-associated (baseline) samples from both groups, while the Day 14 CHX samples largely formed a separate cluster, although three samples clustered with the gingivitis group.

We also performed a separate PCoA on samples from six subjects for whom Day 0 (health), Day 21 (gingivitis), and Day 35 (Day 14 of CHX-treatment) data were available. The results show that while gingivitis was associated with a shift in microbial profile away from health, the use of CHX did not restore baseline composition but shifted it into a new state (Supplementary Figure 1).

### Microbial shift in the prevention sub-study

**Figure 7.**
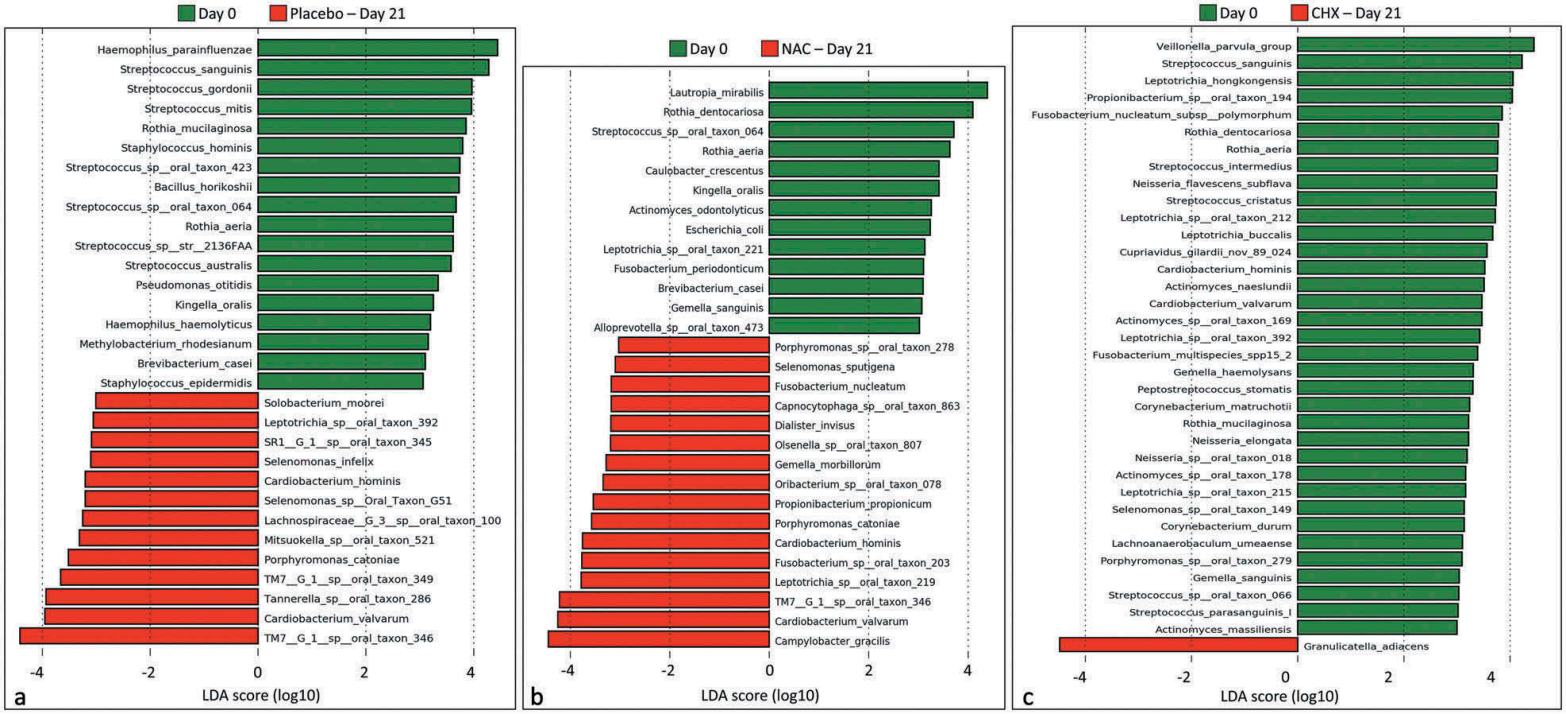
Differentially abundant species between baseline (Day 0) and endpoint (Day 21) in the prevention sub-study. (a) Placebo group, (b) NAC group, and (c) CHX group. Differences were identified by linear discriminant analysis (LDA) effect size analysis (LEfSe); LDA score ≥3. NAC: *N*-acetyl cysteine; CHX: chlorhexidine.

Species associated with healthy gingiva (Day 0) and those enriched on Day 21 in the three groups of the prevention sub-study are shown in Figure 7(a–c). In the placebo group, 13 species were significantly overabundant on Day 21 (full-blown gingivitis), while 18 species were associated with health (Day 0). In the NAC prevention group, 16 bacterial species were found to be enriched on Day 21 (full-blown gingivitis), 4 of which are common with the placebo group: TM7 G1 sp. oral taxon 346, *Cardiobacterium valvarum, Cardiobacterium hominis*, and *Porphyromonas catoniae*. Other top, gingivitis-associated species identified in either group included, among others, *Campylobacter gracilis*, TM7 G1 sp. oral taxon 349, *Tannerella* sp. oral taxon 286, *Leptotrichia* sp. oral taxon 219, and *Fusobacterium* sp. oral taxon 203. Healthy gingiva-associated bacterial species, i.e. those enriched on Day 0 in the placebo or/and NAC groups, were *Haemophilus parainfluenzae, Lautropia mirabilis, Kingella oralis, Rothia mucilaginosa, Rothia dentocariosa, Rothia aeria, Brevibacterium casei*, and several *Streptococcus* spp., among others. The use of CHX for prevention, on the other hand, was associated with depletion of a mix of 35 gingivitis- and health-associated species, while it selected for only one species (*G. adiacens*)

Results of differential abundance analysis at the genus and phylum for the prevention sub-study are presented in Supplementary Figure 2. *Firmicutes* was a health-associated phylum, whereas *Saccharibacteria* (TM7) and SR1 were identified as gingivitis-associated. At the genus level, *Haemophilus, Streptococcus, Rothia, Kingella, Lautropia*, and *Brevundimonas* were found to be associated with gingival health. Gingivitis-associated bacterial genera identified based on analysis of the placebo and NAC prevention groups (i.e. groups that developed severe gingivitis) included *Fusobacterium*, TM7 G1/G6, *Cardiobacterium, Campylobacter*, SR1 G1, *Tannerella, Parvimonas, Selenomonas*, and *Lachnospiraceae* G3; using a less conservative LDA score (e.g. 2.5), additional TM7 genera (G3 and G4) were also found to be associated with gingivitis. Prevention with CHX enriched for *Granulicatella*, while it resulted in a decrease in the relative abundances of a mix of eight gingivitis- and health-associated genera.

### Microbial shifts in the treatment sub-study

**Figure 8.**
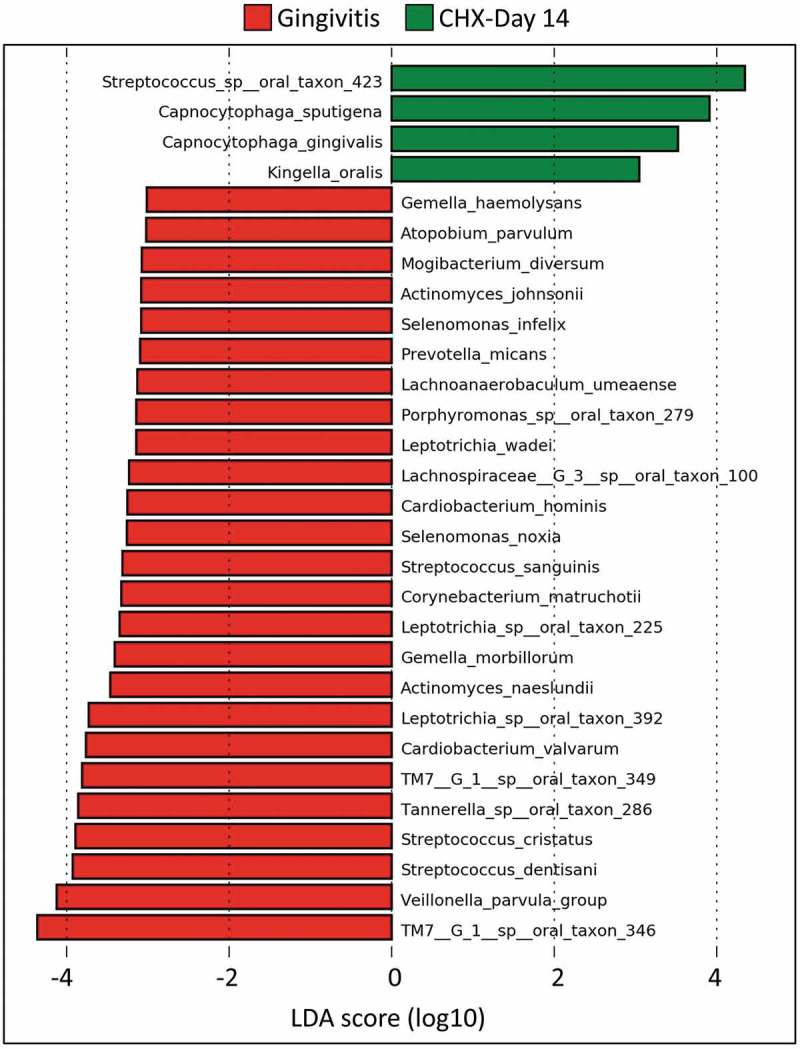
Differentially abundant species between baseline (gingivitis) and endpoint (Day 14) in the chlorhexidine (CHX) treatment group. Differences were identified by linear discriminant analysis (LDA) effect size analysis (LEfSe); LDA score ≥3. No differences were identified for *N*-acetyl cysteine.

Treatment of experimental gingivitis with NAC did not result in significant microbial changes at any taxonomic level. In contrast, CHX treatment resulted in dramatic changes. At the species level, the use of CHX was associated with a reduction in the relative abundances of a mix of 25 gingivitis- and health-associated species and enrichment of 4 species (Figure 8). At the phylum level, it resulted in a decrease in abundance of Phyla TM7, SR1, and *Actinobacteria* (Supplementary Figure 3A). At the genus level, it was associated with a decrease in the relative abundances of 14 genera (again a mix of gingivitis- and health-associated) and an increase in that of only 1 genus, *Capnocytophaga* (Supplementary Figure 2B).

## Discussion

Using prevention and treatment sub-studies, we were able to simultaneously characterize the microbiome shifts associated with transition from health to gingivitis as well as those induced by the use of NAC and CHX mouthwashes. Consistent with results from the parent clinical study [23], the 1.25% NAC mouthwash had modest effectiveness in both prevention and treatment of gingivitis. This was reflected in the microbiological findings as well, since the microbial changes observed in the NAC prevention group were similar to those in the placebo group and using NAC as a treatment resulted in no microbiome shifts. The NAC groups thus provided an additional opportunity to identify health- and gingivitis-associated microbiota. As expected and consistent with the literature [40,41], 0.2% CHX was very effective in both sub-studies, although it did not entirely prevent gingivitis or result in complete remission, similar to what have been shown by others [42]. Although stringent measures were taken to maximize compliance with using the mouthwashes, some lack of compliance cannot be totally excluded and may have accounted for the observed suboptimal effect for CHX. As discussed below, it was evident that CHX mediated its action by indiscriminate antimicrobial actions rather than selectively targeting gingivitis-associated taxa or enriching health-associated ones.

Results from this study showed striking similarity with those of the studies by Huang et al. [12,13]. Four of the genera we identified as significantly overabundant in health (namely *Rothia, Lautropia, Streptococcus*, and *Haemophilus*) were, in addition to *Actinomyces*, the five health-associated genera identified by Huang et al. and used to calculate the microbial index for gingivitis (MiG27) [12]. In fact, *Actinomyces odontolyticus* was also found in our study to be associated with health. Therefore, results from both studies discount the role of *Actinomyces* in gingivitis suggested by some early culture studies [5,6,8]. There were also significant similarities in terms of the disease-associated microbiota; out of 22 gingivitis-associated genera Huang et al. identified and used for calculation of the MiG27, 8 were found to be enriched in gingivitis in our study, namely *Fusobacterium*, TM7 genera, *Tannerella, Selenomonas, Lachnospiraceae*, the SR1 genus, *Campylobacter*, and *Oribacterium*. The association was particularly evident for TM7, with four genera – and the entire phylum – being significantly overabundant in gingivitis. In fact, TM7 taxa were also on top of those that were depleted by the use of CHX in the treatment sub-study. Unlike the study by Huang et al., however, we found that particular species within each genus – with the exception of *Rothia* and *Streptococcus* – accounted for the associations, e.g. TM7 G1 sp. oral taxon 346/349, *C. valvarum, C. gracilis*, and *Tannerella* sp. oral taxon 286 on the top of gingivitis-associated taxa, and *L. mirabilis, H. parainfluenzae*, and *K. oralis* on the top of health-associated species. Furthermore, certain species, like those of *Leptotrichia*, showed association with gingivitis at the species level, but not at the genus level. Another contradiction with the study by Huang et al., as well as some older studies [6–8], is that we did not find *Treponema* to be associated with gingivitis at neither the genus nor the species level.

In contrast with results from *in vitro* studies [17–21], our 1.25% NAC mouthwash was not found to induce significant subgingival microbiome changes: differences between baseline and endpoint in the prevention sub-study were largely similar to those in the placebo group, while there were no differences in the treatment sub-study. This was also evident from the PCoA plots in which samples from the NAC group co-clustered with those from the placebo in the prevention sub-study, and with baseline samples in the treatment sub-study. A major methodological difference that probably accounts for this contradiction is that growing biofilms in *in vitro* studies have been exposed to NAC throughout the experiment, rather than to pulses that simulate using a mouthwash in a clinical trial. Nevertheless, a recent study that assessed the effect of NAC (at concentrations of 0.1%, 1%, and 10%) on multispecies oral biofilm derived from supragingival plaque found that although NAC reduced bacterial biomass and adherence, it did not affect microbial composition and ecology even at the highest concentration tested [22], which is consistent with our finding. Noteworthy, NAC has an unpleasant taste that places a limitation on the maximum concentration that can be used in a mouthwash. In our parent study, we found 1.25% to be the highest tolerable concentration, after adding flavour enhancers.

CHX, on the other hand, had a dramatic effect on both biomass as well as microbial composition. The use of CHX in both sub-studies was associated with significant reduction in dental plaque and a sharp drop in microbial diversity with significant decrease in relative abundances of a mix of health-associated and gingivitis-associated species, indicating a largely indiscriminate antibacterial action. Indeed, CHX is known to be a potent broad antimicrobial [43]. In a five-species *in vitro* oral biofilm, Shapiro et al. showed that exposure to 0.2% CHX resulted in near-complete eradication of viable cells [44]. However, our results show that a number of taxa were enriched with the use of CHX suggesting they may be less sensitive than the others. In fact, McBain et al. [45] in testing CHX against 10 oral bacteria found a number of them including *Lactobacillus rhamnosus, Neisseria subflava*, and *F. nucleatum* to be comparatively less sensitive. Consistently, the latter species showed an increase in relative abundance with use of CHX in our both sub-studies (Figures 3 and 4), which may explain the persistence of some level of gingivitis in some subjects. Obviously, more work is required to understand the microbial shifts associated with use of CHX, preferably using an *in vitro* microbiome model that eliminates the effect of clinical variables.

In conclusion, our results are largely consistent with recent studies on the microbiome of gingivitis; they substantiate the evidence for the polymicrobial nature of the disease and suggest specific subgingival species are involved. However, it remains a challenge to identify which of the microbial changes observed in association with gingivitis represent driver events and which ones are just passenger events. This needs to be explored in carefully designed clinical/experimental studies in the future. Our results also demonstrate the effectiveness of CHX in prevention/treatment of gingivitis, and that such effectiveness is attributed to a non-specific antimicrobial action, resulting in low biomass, rather than selective targeting of the pathogenic species. NAC, on the other hand, did not prove to be a promising mouthwash, at least at the concentration tested.
