# Supplementary material for: Subgingival microbiome of experimental gingivitis: shifts associated with the use of chlorhexidine and N-acetyl cysteine mouthwashes
Source: J Oral Microbiol. 2019 Jun 24;11(1):1608141. doi: 10.1080/20002297.2019.1608141 (PMC6598494; doi:10.1080/20002297.2019.1608141)
Supplement: Supplemental Material [file ZJOM_A_1608141_SM2983.zip › supplemental data/Suppl. figures and tables - R1.docx]

**Subgingival microbiome of experimental gingivitis: shifts associated with use of chlorhexidine and N-acetyl cysteine mouthwashes**

Ahlam Al-Kamel^1^, Divyashri Baraniya^2^, Wadhah Abdulnaser Al-Hajj^1,3^, Esam Halboub^4^, Saleem Abdulrab^5^, Tsute Chen^6,^ Nezar Noor Al-Hebshi^2*^

**Supplementary materials**

**Supplementary table 1**. Characteristics of the study subjects - prevention sub-study.

| **Variables** | **Subcategories** | **Placebo (n=10)** | **NAC**  **(n=10)** | **CHX**  **(n=10)** | **P value** |
| --- | --- | --- | --- | --- | --- |
| Gender | Females | 7 (70) | 9 (90) | 6 (60) | 0.303^†^ |
|  | Males | 3 (30) | 1 (10) | 4 (40) |  |
| Faculty | Dental | 6 (60) | 10 (100) | 9 (90) | 0.134^†^ |
|  | Medical | 2 (20) | 0 (0) | 1 (10) |  |
|  | Engineering | 2 (20) | 0 (0) | 0 (0) |  |
| Study Level | Second | 4 (40) | 2 (20) | 4 (40) | 0.845^†^ |
|  | Third | 1 (10) | 1 (10) | 1 (10) |  |
|  | Fourth | 2 (20) | 1 (10) | 2 (20) |  |
|  | Fifth | 3 (30) | 6 (60) | 3 (30) |  |
| Age, median (IQR) | | 23 (21-23) | 23 (22-23) | 21.5 (20-23) | 0.340^*^ |

NAC: N-acetyl cysteine; CHX: Chlorhexidine. ^†^ Chi-Square test; * Kruskal Wallis test; IQR: Inter-quartile range.

**Supplementary table 2.** Characteristics of the study subjects - treatment sub-study.

| **Variables** | **Subcategories** | **CHX**  **(n=10)** | **NAC**  **(n=10)** | **P value** |
| --- | --- | --- | --- | --- |
| Gender | Males | 3 (30) | 5 (50) | 0.361^†^ |
|  | Females | 7 (70) | 5 (50) |  |
| Faculty | Dental | 7 (70) | 9 (90) | 0.119^†^ |
|  | Medical | 3 (30) | 0 (0) |  |
|  | Engineering | 0 (0) | 1 (10) |  |
| Study Level | Second | 2 (20) | 3 (30) | 0.94^†^ |
|  | Third | 3 (30) | 3 (30) |  |
|  | Fourth | 2 (20) | 2 (20) |  |
|  | Fifth | 3 (30) | 2 (20) |  |
| Age, median (IQR) | | 22.5 (21-23) | 23 (22.75-23.25) | 0.631^*^ |

NAC: N-acetyl cysteine; CHX: Chlorhexidine. ^†^ Chi-Square test; * Mann Whitney U test; IQR: Inter-quartile range.

**Supplementary table 3.** Changes (mean (SD)) in gingival health indices in the prevention sub-study

| **Variable** | **Placebo**  **(n=10)** | **NAC**  **(n=10)** | **CHX**  **(n=10)** |
| --- | --- | --- | --- |
| Change in PI (21^st^ Day – Baseline) | 2.12 (0.38) | 1.90 (0.41) | 0.20 (0.26) ^†*^ |
| Change in GI (21^st^ Day – Baseline) | 1.84 (0.08) | 1.52 (0.28)^*^ | 0.44 (0.22)^†*^ |
| Change in PBI (21^st^ Day – Baseline) | 1.96 (0.42) | 1.53 (0.60) | 0.41 (0.19)^†*^ |

NAC: N-acetyl cysteine; CHX: Chlorhexidine. Significance of between-group differences were sought 1-way ANOVA followed, whenever significant, with pairwise comparisons using Bonferroni correction. ^†^ Change statistically significantly different from the placebo group; ^*^ Change statistically significantly different from the other group.

**Supplementary table 4.** Changes (mean (SD)) in gingival health indices in the treatment sub-study

| **Variable** | **CHX**  **(n=10)** | **NAC**  **(n=10)** | **P value^†^** |
| --- | --- | --- | --- |
| Change in PI (14^th^ day – Baseline) | - 1.89 (0.52) | - 0.1 (0.32) | < 0.001 |
| Change in GI (14^th^ day – Baseline) | - 0.97 (0.38) | - 0.24 (0.37) | < 0.001 |
| Change in PBI (14^th^ day – Baseline) | - 1.35 (0.21) | - 0.26 (0.66) | < 0.001 |

NAC: N-acetyl cysteine; CHX: Chlorhexidine. – denotes a decrease relative to the previous baseline measurement. ^†^ Between-group differences, independent t-test.


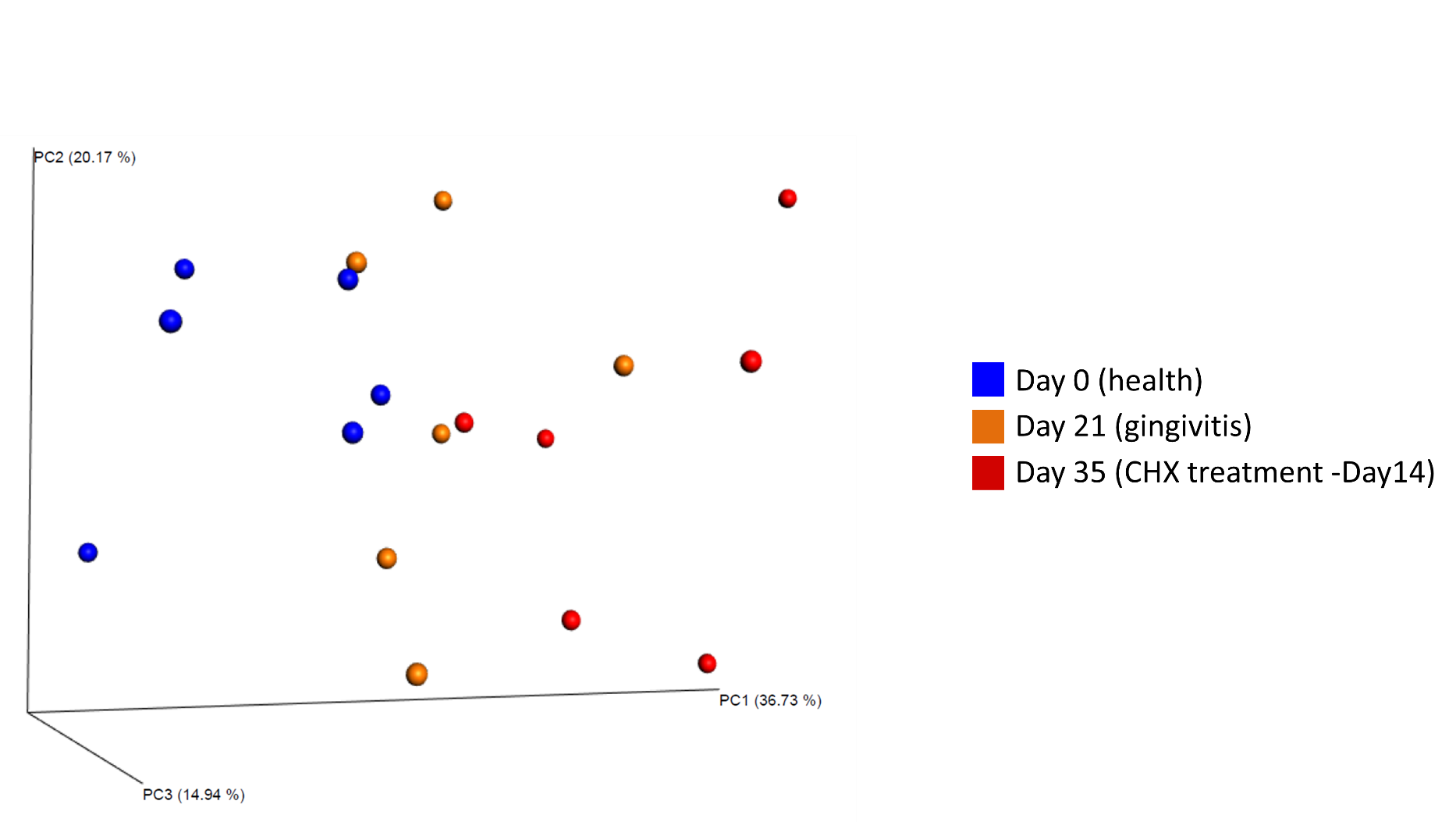


**Supplementary Figure 1**. Principle component (PCoA) analysis on a subset of samples to assess whether or not treatment of gingivitis with chlorhexidine (CHX) restores baseline (Day 0) microbial composition. Samples were clustered based on -Jaccard distance matrix. As shown, treatment with CHX shifted microbial composition to a new state different from that at baseline.


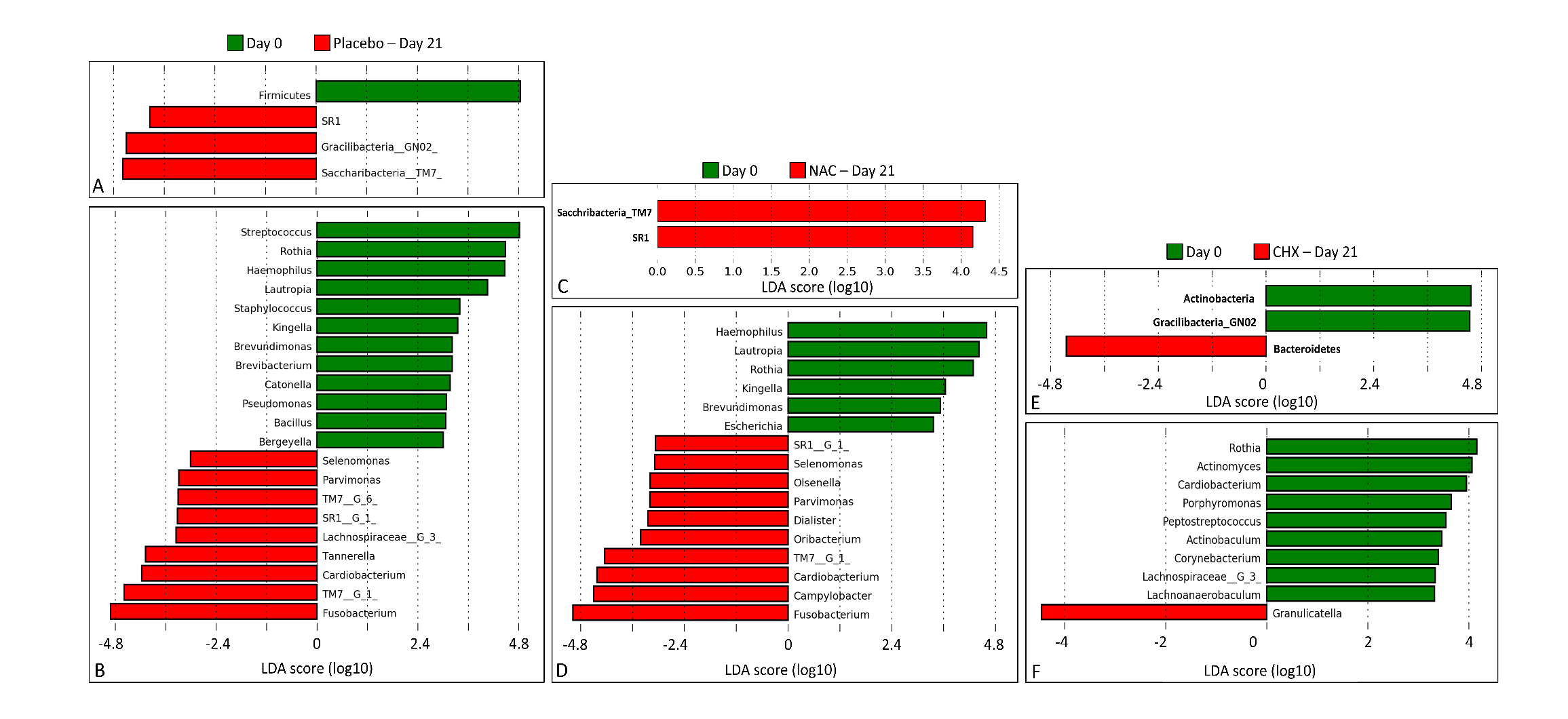
**Supplementary Figure 2**. Differentially abundant phyla (A, C & E) and genera (B, D & F) between baseline and Day 21 (Prevention sub-study) as identified by linear discriminant analysis (LDA) effect size analysis (LEfSe); LDA score ≥ 3. NAC: N-acetyl cysteine; CHX: Chlorhexidine.


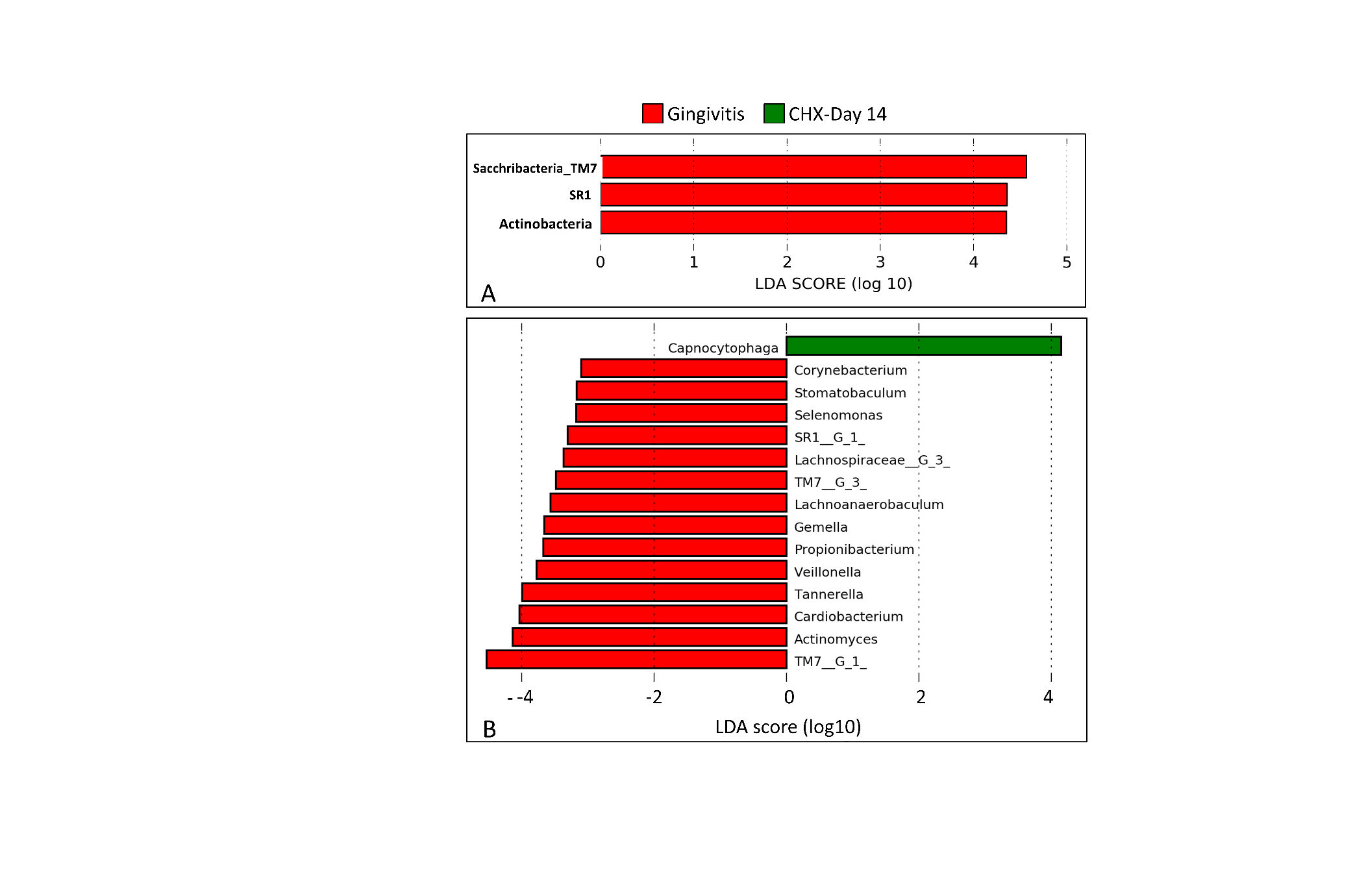


**Supplementary Figure 3**. Differentially abundant phyla (A) and genera (B) between gingivitis and Day 14 (Treatment sub-study) as identified by linear discriminant analysis (LDA) effect size analysis (LEfSe); LDA score ≥ 3. CHX: Chlorhexidine. No differences were identified for N-acetyl cysteine.
